# Advanced Development of Molecularly Imprinted Membranes for Selective Separation

**DOI:** 10.3390/molecules28155764

**Published:** 2023-07-30

**Authors:** Jiahe Chen, Maobin Wei, Minjia Meng

**Affiliations:** 1 College of Physics, Jilin Normal University, 1301 Haifeng Street, Siping 136000, China; veniceb1chen@163.com; 2 School of Chemistry and Chemical Engineering, Jiangsu University, Zhenjiang 212013, China

**Keywords:** molecular imprinting membrane, selective separation, flux, permselectivity, high-density recognition sites

## Abstract

Molecularly imprinted membranes (MIMs), the incorporation of a given target molecule into a membrane, are generally used for separating and purifying the effective constituents of various natural products. They have been in use since 1990. The application of MIMs has been studied in many fields, including separation, medicine analysis, solid-phase extraction, and so on, and selective separation is still an active area of research. In MIM separation, two important membrane performances, flux and permselectivities, show a trade-off relationship. The enhancement not only of permselectivity, but also of flux poses a challenging task for membranologists. The present review first describes the recent development of MIMs, as well as various preparation methods, showing the features and applications of MIMs prepared with these different methods. Next, the review focuses on the relationship between flux and permselectivities, providing a detailed analysis of the selective transport mechanisms. According to the majority of the studies in the field, the paramount factors for resolving the trade-off relationship between the permselectivity and the flux in MIMs are the presence of effective high-density recognition sites and a high degree of matching between these sites and the imprinted cavity. Beyond the recognition sites, the membrane structure and pore-size distribution in the final imprinted membrane collectively determine the selective transport mechanism of MIM. Furthermore, it also pointed out that the important parameters of regeneration and antifouling performance have an essential role in MIMs for practical applications. This review subsequently highlights the emerging forms of MIM, including molecularly imprinted nanofiber membranes, new phase-inversion MIMs, and metal–organic-framework-material-based MIMs, as well as the construction of high-density recognition sites for further enhancing the permselectivity/flux. Finally, a discussion of the future of MIMs regarding breakthroughs in solving the flux–permselectivity trade-off is offered. It is believed that there will be greater advancements regarding selective separation using MIMs in the future.

## 1. Introduction

The separation process is the most crucial part in the chemical industry, since it directly determines the quality and cost of products [1,2]. The methods often used for separation are distillation [3], extraction [4], adsorption [5], crystallization [6], chromatography [7], and membrane separation [8]. In particular, membrane-separation technology (MST) is an advanced separation technology worthy of in-depth study due to its efficient separation performance and environmental friendliness [9], since it usually operates under mild conditions with no phase changes and relatively low energy consumption [10]. After the pioneering experiments using pig bladder for permeation by Abble Nollet in 1748 [11], over-filtration was first conceived by A. Schmidt in 1861. Researchers have since continued to innovate in the fabrication of artificial membranes, and membrane technologies such as microfiltration [12], ultrafiltration [13], nanofiltration [14], reverse osmosis [15], dialysis [16], and electrodialysis [17] have emerged. Membrane-separation material is a kind of medium which plays the role of molecular-level separation and filtration [18]. Membranes are like separation screens for large and small molecules, and the desired molecular-level separation is achieved by selecting a membrane with an appropriate pore size [19]. Therefore, membrane separations primarily rely on disparities in molecule size. The introduction of molecular-recognition sites is essential for enhancing the selectivity of synthetic membranes, as they can effectively distinguish between target molecules and others. The introduction of such molecular-recognition sites into synthetic membranes preferentially incorporates the target molecules into the membrane; these molecules can play an important role in the transport of specific substrates, such as chiral separation and drug purification [20]. Molecular imprinting is a method used to introduce molecular-recognition sites into polymeric membranes [21]. Molecular-imprinting technology (MIT) is known as an “antibody mimic” method for the design of molecularly imprinted polymers (MIPs) by making customized binding sites that match the template molecule in shape, size, and functional group, with predetermined selectivity and high affinity [22,23]. Through the combination of MIT with membrane-separation technology, molecularly imprinted membranes (MIM) have achieved significant advancements in the fields of molecular-specific recognition, biomacromolecule separation, and chiral-compound separation.

The MIT was first established by Wulff in the early 1970s [24]. The MIPs are composite materials with molecular memory function based on the “lock-and-key” mechanism of the copolymerization process. The specific synthesis process is always as follows: the MIT usually contains two molecular-imprinting approaches, covalent and noncovalent molecular imprinting. In covalent molecular imprinting, the first proper functional monomer should be selected to form covalent interactions with a template molecule [25]. In non-covalent molecular imprinting, the interaction between the functional monomer and the template molecule is non-covalent, specifically through hydrogen bonding [26,27]. The next step in the process is polymerization, which involves a solvent–template–monomer–cross-linker–initiator system. This system enables the formation of highly cross-linked and robust three-dimensional (3D) materials. In the final step, a special solvent is used to extract the template molecule, resulting in the desired three-dimensional MIP. This MIP possesses imprinted cavities that are complementary to the template molecule in terms of size, shape, and chemical groups, allowing high selectivity in molecular recognition. Of these approaches, the non-covalent molecular imprinting technology is the currently the most commonly used due to its advantages, such as its simple synthesis process and its applicability with a wide range of templates and most functional monomers [28].

As described above, MIT is considered one of the most simple methods with which to incorporate molecular0recognition sites into polymeric membranes. In a recent review, Yang et al. comprehensively summarized MIMs, including their methods of synthesis, characterization methods, performances, mechanisms, and applications, thereby providing an accurate and complete understanding of MIMs [29]. Furthermore, MIMs have significant applications in selective separation, especially for chiral separation and extraction and the separation of Chinese medicinal compounds. Nevertheless, the flux and permselectivity of these membranes are insufficient for industrial applications. To achieve higher flux and permselectivity, there is a need for MIMs possessing an increased number of effective recognition sites and greater porosity [30,31]. However, conventional MIMs often suffer from a decrease in the number of effective recognition sites due to the embedding of effective imprinting sites, which leads to weak separation efficiency in MIMs, to some extent. This review is intended to provide a comprehensive assessment of MIMs, including their development and various preparation methods used in this membrane technology as a selective separation material, to focus on the flux/permselectivity-trade-off relationship in the separation process, to analyze the rules of the mass-transfer mechanism, to show the important parameters of regeneration and antifouling performance, and to highlight currently emerging MIMs. The last part of the review discusses the future outlook of MIMs.

## 2. Development of Molecular Imprinting Membranes

The use of MIMs began in 1990. Piletsky et al. [32] first reported molecularly imprinted membranes using the “template polymerization” technique for the selective separation of adenosine monophosphate (AMP) and guanosine monophosphate (GMP) by electrodialysis. In 1996, in order to further the selective permeation of adenosine from adenosine–guanosine mixtures, Mathew-Klotz et al. [33] prepared standalone-imprinted polymer films by polymerizing a solution of ethylene glycol dimethacrylate and methyl methacrylate in dimethylformamide (DMF) using 9-ethyladenine as the template and AIBN as the initiator. Under a nitrogen atmosphere, the polymerization and film formation were simultaneously performed on a silanized glass slide at temperatures of 65–70 °C. During in situ polymerization, the polymerization temperature must be kept constant [34]. Any abrupt increase in polymerization temperature may lead to the formation of pinholes in the membrane due to the foaming of the monomers.

**Figure 1:**
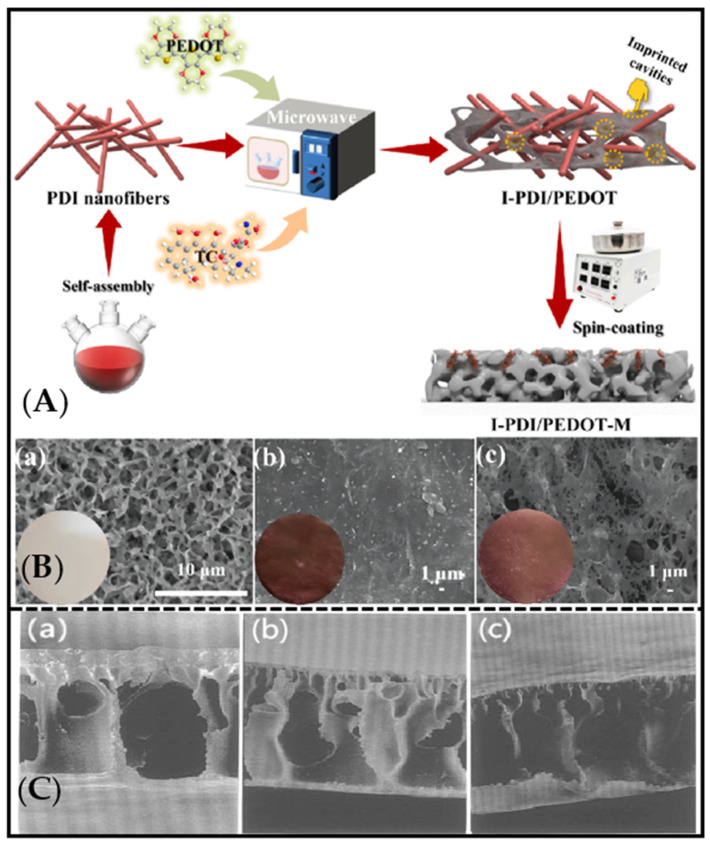
(**A**) Synthesis-process schematic of I-PDI/PEDOT-M. Reproduced with permission from ref [47]. Copyright 2023 Elsevier. (**B**) SEM images of (**a**) PVDF-M, (**b**) PDI-M, and (**c**) I-PDI/PEDOT-M. Reproduced with permission from [47]. Copyright 2023 Elsevier. (**C**) SEM photographs of the cross section of the poly(acrylonitrile-co-acrylic acid)-based MIM coagulated at (**a**) 10 °C, (**b**) 23 °C, and (**c**) 30 °C. Reproduced with permission from [35]. Copyright 1997 Langmuir.

In 1997, Kobayashi et al. [35] reported the new imprinted membranes membranes of theophylline (THO) using the phase inversion method. In this study, the poly(acrylonitrile-co-acrylic acid)-based MIM was prepared by phase-inversion precipitation with the THO molecule as the template molecule and the DMSO copolymer as the functional monomer. The selectivity was proven by using the structural analogy of caffeine (CAF), which showed the selectivity owing to the hydrogen bonding between THO molecules and poly(acrylonitrile-co-acrylic acid)-based MIM. Moreover, Kobayashi et al. first carefully discussed the influence of the coagulation process on the molecular-imprint characteristics. It was shown that the formation of THO-imprinted sites was strongly influenced by the coagulation temperature of P(AN-co-AA) during the phase-inversion process. Figure 1C shows the SEM photographs of a cross-section of the poly(acrylonitrile-co-acrylic acid)-based MIM with different coagulating temperatures at (a) 10 °C, (b) 23 °C, and (c) 30 °C. Table 1 also shows the best selectivity factor obtained at the coagulating temperature of 10 °C for the poly(acrylonitrile-co-acrylic acid)-based MIM. Moreover, the IR results indicated that the effective adsorption of the THO was due to the non-dimerized COOH segments in the membrane interacting with the THO molecules through hydrogen bonds. This research first studied the effect of the coagulation-bath temperature on the phase-inversion imprinting process. However, the recognition sites of these MIMs tend to have a weak affinity with the template molecule due to the membrane-solubilization phenomenon, resulting in poor selectivity [36]. Therefore, in order to improve this weak selectivity, a phase-inversion method was developed by embedding pre-synthesized MIP particles into the membrane matrix [29].

Next, the molecular-imprinting composite membranes appeared by filling an already synthesized molecularly imprinted polymer between the two layers of membranes, in which the polymer particles have a variety of forms, including blocks, rods, gels, granules, nanoparticle-surface coatings, etc. [37,38,39,40]. Due to the structural characteristics of molecularly imprinted polymer particles with large specific surface areas, the adsorption performance of the template molecule is better, but because the molecularly imprinted polymer particles are filled in a “filter cake” layer, the dense particle layer is prone to high resistance to the mass-transfer process and to reduced flux [41]. In 2002, Lehmann et al. [42] fixed the MIPs between two layers of polyamide membranes to obtain a molecularly imprinted composite membrane to achieve the selective recognition and purification of the target-molecule BFA. However, owing to the defects of traditional MIPs in post-processing (the grinding process changes the particle structure and surface morphology of molecularly imprinted polymers, affecting their selectivity), the application range of these molecularly imprinted membranes is limited. Moreover, thick symmetric MIM also have rather low membrane fluxes and large mass-transfer resistance [43].

Subsequently, alternative molecularly imprinted composite membranes were prepared with a thin MIP-layer grafting or casting to the surface of a stable support membrane [44,45]. In the early stage, the heterogeneous photo-grafting approach was commonly used to design MIMs with thin MIP layers on the surfaces of matrix membranes due to the lower energy of UV radiation and the simple operation. In 1998, Hong et al. [11] reported a new type of ultrathin-film-composite membrane for the selective separation of theophylline using the photopolymerization method, in which a MAA/EDMA mixture was placed on top of an asymmetric 20-nanometer-pore alumina membrane. The ultrathin film’s composite membrane obtained higher permeant fluxes than would have been possible with thick, free-standing membranes. Kochkodan et al. [46] also reported thin-layer molecularly imprinted composite membranes by using the coating of a photo-initiator on the surfaces of PVDF membranes for the selective binding of triazine herbicide desmetryn.

Furthermore, the functional molecular imprinting of layers on the surfaces of membrane materials by surface polymerization [48], surface grafting [49], interfacial condensation [50] or coating on the basis of existing base membranes (membrane matrices with good flux and porous structures, like commercial microfiltration or ultrafiltration membranes) [51]. The general preparation steps for molecularly imprinted composite membranes are usually as follows [52]: (1) the substrate material is immersed in a mixed solution containing template molecules, monomers, and initiators; (2) polymerization is initiated on the membrane surface; (3) the elution of template molecules to obtain molecularly imprinted composite membranes. More recently, the surface modification [53] method has been commonly used to prepare molecularly imprinted composite membranes [54]. Lee et al. successfully prepared highly selective nanocomposite-imprinted membrane materials by compounding various materials (e.g., dendrimers and metal nanomaterials) on the surfaces of glass fibers [55].

Recently, the development of molecular-imprinting technology has gradually matured. The MIP is considered a highly promising production strategy. Preliminary commercial applications of electrochemical sensors, artificial receptors, and catalysts prepared using molecular-imprinting technology have already been put into use, which is of great importance in the fields of drug delivery, disease diagnosis, and, especially, in the production of selective extractive adsorbents. In particular, some commercial MIMs have also been introduced. For example, Schwark et al. developed epitope-imprinted membranes targeting the c-terminal fragment of immunoglobulin G (IgG) heavy chain and used them to purify commercial monoclonal antibodies. However, most MIMs are still in the exploratory stage, and the industrial production of MIMs will be an important area of research in the future.

## 3. Preparation Method for Molecularly Imprinted Membrane

### 3.1. In Situ Polymerization Method

The in situ polymerization method is usually used for synthesizing self-supported MIM [56,57,58]. The template molecule, functional monomer, and crosslinking agent in a certain proportion are mixed to make a casting solution, followed by immersing the supporting membrane into the casting solution to ensure it is fully cross-linked (the process, characteristics, and references in the preparation method mentioned in this paper are shown in Table 2). After the polymerization reaction, the template molecule is eluted to obtain a molecularly imprinted membrane. For example, Sergeyeva et al. [59] prepared a molecularly imprinted membrane using in situ polymerization method as follows. Firstly, a mixture of template molecules (Atrazine), functional monomer (methacrylic acid), cross-linker (tri(ethylene glycol) dimethacrylate and oligourethane acrylate), initiator (1,1′-azobis(cycloxexane carbonitrile), and 50% (*w*/*w*) of the organic solvent were prepared for use. Next, a 60 µm gap between two glass slides was immersed in the mixture and exposed to UV light (*λ*) 365 nm for 1 h. After reaction, an 8 h extraction procedure was carried to remove non-polymerized compounds and the template molecules to finally obtain the porous molecularly imprinted polymer membranes. The advantages of this method are that the preparation process is simple, and that the obtained membrane has strong rigidity and low porosity. However, shortcomings exist in the membrane prepared by this method in terms of its high level of thickness, poor permeability, and difficult diffusion of template molecules out of the imprinted membrane. Since there are certain limitations in the practical application process, appropriate additives should be added to improve the recognition and separation performance of the membrane and increase its permeability [60,61].

### 3.2. Phase-Inversion Method

Phase-inversion process is the most commonly used method for the preparation of MIMs, in which a certain amount of template molecules and functional polymers are dissolved in a suitable solvent, after which they are scraped on support and placed in a coagulation bath or inert gas atmosphere to directly obtain a polymer membrane with molecule-specific recognition [62,63,64]. For example, Zeng et al. [65] fabricated an efficient ion-imprinted membrane with platinum(IV) of template ion via non-solvent-induced phase separation (NIPS) method and realized the selective separation of platinum(IV). Algieri et al. [66] also prepared novel hybrid imprinted membranes for selective recovery of theophylline via phase-inversion method. Cui et al. [67] constructed a degradable imprinted membrane based on cellulose acetate/chitosan hybrid membrane via phase-inversion method and realized the selective separation and recovery of Li+. The advantage of the phase-inversion method is that it can increase the flux of the membrane, and the recognition sites of MIM can be obtained directly in the polymer material without addition of the imprinted molecule [68,69].

**Table 2:** Comparison of MIM preparation methods.

Method	Characteristics	Preparation Process	Ref.
In situ polymerization method	Simple preparation process. The obtained membrane has high rigidity and low porosity, but also high level of thickness, poor membrane permeability, and difficulty in diffusion of template molecules out of the imprinted membrane	The support membrane is immersed in the casting solution made of template molecule, functional monomer, and crosslinking agent, and the template molecules are eluted after the polymerization reaction	[59]
Phase-inversion method	Improves membrane flux and allows the acquisition of MIM-recognition sites directly in polymeric materials without the need for additional imprinted molecules	A certain amount of template molecules and functional polymers are dissolved on the carrier in a suitable solvent and placed in a coagulation bath or an inert gas atmosphere	[65,66,67]
Coating method	It is simple to operate and has good application prospects, but the prepolymer solution must have a suitable concentration	The prepolymer solution is dispersed in the solvent and uniformly dispersed in the substrate film. The composite layer is fixed on the surface of the substrate by chemical-crosslinking method	[70,71]
Electrochemical process	Fast preparation speed, controllable thickness of imprinted film can be prepared directly on the electrode surface, low level of film thickness, and solves the problem of contact between the film and sensor interface	Selection and cleaning of the appropriate electrode, preparation of molding fluid, and elution of imprinted molecules after electrochemical polymerization	[72,73]
Sol–gel process	Excellent selectivity, adsorption rate, and kinetic properties	Hydrolysis of precursors, condensation, gelation, and thermal treatment of the sol–gel material after drying	[74,75,76]

### 3.3. Coating Method

The molecularly imprinted membrane is prepared by means of coating, as follows. The prepolymer solution with a suitable concentration was dispersed with a solvent, and then it was uniformly dispersed on the substrate membrane by dip coating or drop coating. The composite layer is fixed on the surface of the substrate membrane by chemical-crosslinking method [77,78]. For example, Li et al. reported a molecular-imprinted membrane by coating cellulose acetate onto a ZrO_2_-modified alumina membrane for the chiral separation. In this research, ZrO_2_-modified channel membrane was first prepared, and it was then immersed in a cast membrane liquid with template molecules of (S)-(+)-mandelic acid, followed by static evaporation, and subsequently immersed in pure water to remove the solvent [70]. Lu et al. [47] first reported the preparation of 1-methyl-2-pyrrolidone (NMP)-induced surface self-corrosion-assisted imprinted perylene diimide/poly-3,4-ethylenedioxythiophene heterojunction photocatalyst anchoring film (I-PDI/PEDOT-M) by rapid spin-coating method. In this study, the imprinted PDI/PEDOT heterojunction photocatalyst (I-PDI/PEDOT) was synthesized and uniformly dispersed in 10 ml NMP solvent. The mixed solution was dropped on the PVDF membrane (PVDF-m). After the spin coating, the membrane was immersed in deionized water and dried (Figure 1A). As shown in Figure 1B, the color of each membrane also changed significantly after rotating the coating on the surface of PVDF-M. This method is simple to operate and has good application prospects. However, it should be noted that the prepolymer solution should have an appropriate concentration. Otherwise, when the concentration of prepolymer is excessively low, its viscosity and surface tension are reduced, making it difficult for the prepolymer solution to be uniformly coated on the surface of the base film. If the concentration is excessively high, the thickness of the functional layer of the composite-imprinting film is affected. Fujikawa et al. reported free-standing ultrathin films of metal oxides via a combination of spin coating and molecular imprinting [71]. The structure of the free-standing and shape-selective MIM was filled with molecule channels by random linking of individual cavities for template molecules. The research indicated that the amount of template molecules was strongly influenced the porosity of the imprinted membrane [71].

Interestingly, in the case of the 4-(phenylazo)benzoic acid-imprinted membrane, when the size of filtrated molecules increased, the concentration of the filtrated molecules decreased. In other words, the selective channel determined by the size of individual template molecule can precisely recognize the size of filtrate molecules. This study also showed that when the thickness of the coating-imprinted membrane is 30 nm, the filtration molecules migrate in the channel via random collision. When the membrane becomes thinner, the length of the channel reduces, which can facilitate highly efficient filtration.

### 3.4. Electrochemical Process

**Figure 2:**
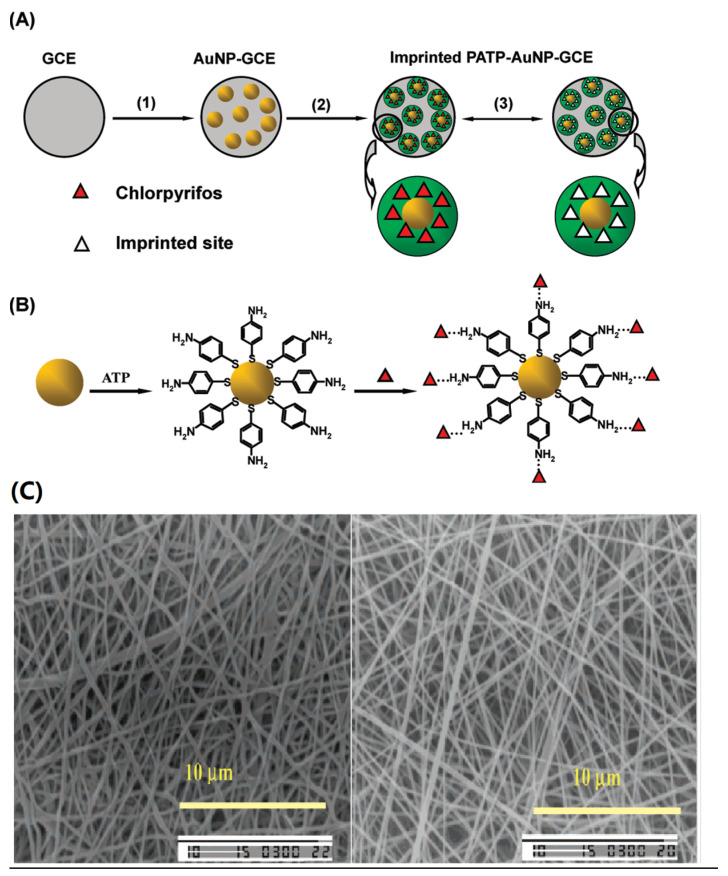
(**A**) Preparation steps of the imprinted PATP-AuNP-gc electrode: (1) electrodeposition of AuNPs on the surface of the gcelectrode; (2) electropolymerization of ATP on the surface of the AuNP-gc electrode; (3) removal/rebinding of CPF on the imprinted sites of the imprinted PATP-AuNP-gc electrode. Reproduced with permission from [72]. Copyright 2010 American Chemical Society; (**B**) Schematic illustrations for the adsorption of the ATP molecule at the AuNP surface and the furtherself-assembly of CPF at ATP-modified AuNP-gc electrode. Reproduced with permission from [72]. Copyright 2010 American Chemical Society; (**C**) 2,4-D imprinted nanofiber after template removal and non-imprinted nanofibers 5000× magnification. Reproduced with permission from [81]. Copyright 2006 American Chemical Society.

Electrochemical methods are well-established analytical tools that are mostly used for the preparation of sensitive imprinted membranes for sensors [79]. In 2003, Blanco-López’s group reported a voltametric sensor for vanillylmandelic acid (VMA), prepared by spin coating MIP layer on the surface of a glassy carbon electrode (GCE) with a monomer mixture (template, methacrylic acid, a cross-linking agent, and solvent), followed by in situ photo-polymerization. The target molecule was detected due to its appropriate pore size and pore density for the analyte diffusion towards the electrode surface [80]. Nevertheless, the membrane obtained suffered from instability and difficulties in controlling its thickness. Furthermore, the imprinted membranes were unable to selectively bind analytes in aqueous systems, severely restricting their potential applications. Expanding on these findings, Xie et al. presented a surface-molecular-self-assembly strategy that utilizes gold nanoparticle (AuNPs)-modified glassy carbon (GC) electrodes for electro-polymerization, enabling molecular imprinting on polyaminothiophenol (PATP) films. This approach is employed for electrochemical detection of the pesticide chlorpyrifos (CPF) (Figure 2A,B) [72]. In that research, employing an AuNP-modified electrode with a larger surface area resulted in a substantial increase in the ratio of imprinted sites and the overall quantity of effective imprinted sites. This advancement not only significantly enhanced the sensitivity and selectivity of CPF analysis but also achieved excellent repeatability. The self-assembled monolayer (SAM) has been widely used in sensors due to its excellent stability and integration with biomolecular electronic devices. Yuan et al. [73] reported a novel molecularly imprinted sensor (MIS) successfully combining self-assembly, electro-polymerization, and molecular-imprinting technologies. The PtNPs have highly specific surface areas and electro-catalytic activities, accommodating more imprinted sites and promoting electron transfer in the electro-chemical sensor. The SAM of 17-estradiol and 6-mercaptonicotinic acid can drive the occurrence of in situ electro-polymerization at the PtNP/GCE surface. Capacity tests clearly demonstrated that the MIS had high selectivity and sensitivity towards 17-estradiol. 

### 3.5. Sol–gel Process

Sol–gel is a procedure known for the transformation of a system from a liquid solute (the colloidal suspension of particles) into solid gel [74]. The typical sol–gel processes typically involves hydrolysis of precursors, condensation, gelation, and thermal treatment of a sol–gel material after drying [75]. Queirós et al. reported a sol–gel imprinted sensing membrane by incorporating the template of microcystin-LR into the sol–gel phase; when the mixture is hydrolyzed, the substrate is polymerized in a three-dimensional network. Eventually, the template was removed from the polymer, leaving empty spaces. The proposed device is faster and less costly than previous methods reported in the literature for MCT determination in water [74].

Subsequently, an alternative sol–gel imprinting method was developed for preparing MIMs. This methodology combines the non-hydrolytic sol–gel (NHSG) process with molecular-imprinting technique. The NHSG technique eliminates the need for prolonged aging and high-temperature drying steps. Additionally, it minimizes or eliminates the generation of water during the synthesis process, which helps prevent cracking and shrinking of the gelatinous material observed in hydrolytic sol–gel methods. As a result, the NHSG process preserves the binding sites and enhances the selectivity of the imprinted material. Meng et al. [76] reported new molecular-imprinted alumina membranes for selective separation of gentisic acid (GA) from salicylic acid using NHSG imprinting method with room-temperature ionic liquid (RTIL) used as the pore template. The results showed that the incorporation of RTIL can greatly increase the porosity, flux, and recognition ability, as well as further improving the selectivity of the imprinted membrane to GA.

## 4. Important Parameters in the Separation Application of the MIM

### 4.1. Selective Separation

#### 4.1.1. Permselectivity/Flux

The permselectivity and flux [82] are the two most important parameters of specificity in membrane separation, and they often have a trade-off relationship. The enhancement of the flux through the MIM usually leads to a simultaneous reduction in permselectivity and vice versa. It is important to enhance both of the two key factors for MIMs to be applicable in various industries. An ultimate aim of membranologists is to simultaneously enhance not only permselectivity, but also flux [83,84].

Huang et al. first reported a molecular-imprinted Al_2_O_3_ nano-channel membrane modified with ZrO_2_ coated with cellulose acetate (CA) containing template molecules for the chiral separation of (D,L)-lactic acid. In this research, infiltration experiments were studied in detail to reveal the relationship between the flux and the permselectivity of the membrane using the (D,L)-lactic acid as the feed solution. Some interesting findings were obtained, as follows. (1) The flux of the imprinted membrane decreased with the increasing CA concentration (10 wt%, 15 wt%, and 20 wt% in the casting solution) and test duration. In addition, when the CA concentration was 20 wt%, the MIM exhibited high enantioselectivity at first, while, after a separation of about 430 min, the 15 wt% CA exceeded the separation effect of the CA content of 20 wt%. Moreover, the 10 wt% CA MIM showed the lowest enantioselectivity. This phenomenon was mainly ascribed to the chiral-recognition sites dispersed in the MIM. For the MIM with a CA content of 10 wt%, the chiral-recognition sites were limited in the membrane, decreasing the permselectivity. For the CA content of 15 wt%, the chiral sites were appropriate, and the feed liquid was sequentially identifiable according to each chiral-recognition site, increasing the permselectivity. For the CA content of 20 wt%, there many chiral-recognition sites were observed in the membrane, but the irregularity of the molecular motion also increased, ultimately reducing the selectivity. (2) Compared with the molecularly imprinted cellulose-acetate membrane, the molecular-imprinted cellulose-acetate-composite membrane showed higher flux and efficiency in the separation of lactic acid. This phenomenon may have been due to the fact that the number of recognition sites in the total thickness of the imprinted cellulose-acetate-composite membrane was greater than that in the thin-layer imprinting of the cellulose film.

Compared with previous studies on enantioselective membranes (Table 3), an alternative method for the simultaneous enhancement of permselectivity and flux in membranes is presented. In conclusion, appropriate and abundant selective recognition sites in the MIM are crucial to the enhancement of permselectivity and flux.

Additionally, Yoshikawa et al. [82] pointed out that MIMs with high porosity and high surface area can provide higher flux and permselectivity. Nanofiber membranes were considered the candidate membranes with a porosity of about 80%, whereas a typical film-type membrane has a porosity of only 5–10%. Molecularly imprinted nanofiber membranes (MINM) compete for the title of highest-performing MIM, aiming for the highest flux and selectivity.

For instance, Sueyoshi et al. prepared MIMs from cellulose acetate (CA) and a derivative of optically pure glutamic acid as a print molecule by simultaneously applying MIT and electrospray deposition. The results showed that the membranes were about two orders of magnitude higher than the usual MIMs, while maintaining permselectivity, proving that permselectivity and flux can be simultaneously enhanced.

Subsequently, Gao et al. [90] reported “nanomagnet-inspired” molecularly imprinted nanofiber membranes prepared by using the electrospinning method for the selective separation of luteolin. The authors directly immobilized the functional groups on the nanofiber membrane’s surface so that the imprinted polymerization formed on the surface of the membrane. In their research, biosynthesized manganese nanoparticles were first introduced to provide rich -OH and -COOH groups for anchoring the luteolin, significantly improving the efficiency of the imprinted sites. Moreover, in order to enhance the precision of the recognition sites, the covalent/non-covalent interactions synergistically drove the strong matching of the spatial structure of luteolin. This study endowed the imprinted nanofiber membranes with a stable internal structure to break through the trade-off effect between the flux (1199.14 L m^−2^ h^−1^, 0.1 MPa) and the permselectivity (4.41 and 5.41).

In general, the construction of a molecularly imprinted membrane with effective high-density recognition sites and strong matching of imprinted cavity is of paramount importance in solving the permselectivity/flux trade-off relationship.

#### 4.1.2. Mechanisms for Selective Transport

Membrane separation is possible through the coupling of the template binding to MIP sites in a MIM, with selective transport through the MIM. Early in 2004, Ulbricht first proposed two major mechanisms for selective transport, as follows [91]:

(1) “retarded permeation”

The retarded permeation of a template caused by affinity binding. The transport of other solutes increases in speed until saturation of MIP sites with the template is achieved.

(2) “facilitated permeation”

The permeation is facilitated by the template’s preferential binding due to affinity binding, while transport of the other solutes is slowed.

**Figure 3:**
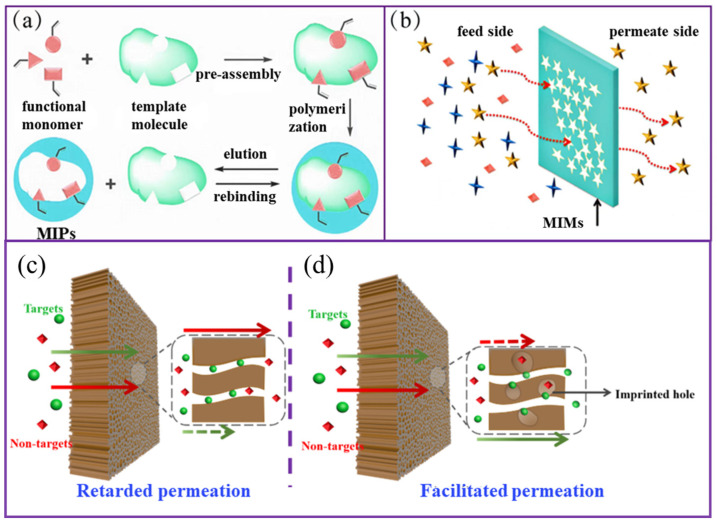
(**a**,**b**) The mechanism diagram of retarded permeation; (**c**,**d**) the mechanism diagram of facilitated permeation.

In case (1), the primary factor influencing the separation efficiency is the binding capacity of the MIPs. Since selectivity arises from specific adsorption, these MIMs can be viewed as membrane adsorbers. In case (2), the target transport can occur via facilitation transport, depending on the membrane structure, as well as the concentration and distribution of MIP sites in the MIM. (Figure 3).

Ulbricht also pointed out that the pore morphologies of MIMs are of major importance in selective transport. Bai et al. [92] reported an upper-surface-imprinted membrane using the magnetic-guidance phase-inversion method for the selective separation of artemisinin. The authors compared the conventional blending-phase-inversion method and magnetic-guidance phase-inversion method to prepare the MIM. The results showed that the MIM prepared by magnetic guidance exhibited a narrower pore-size distribution and more efficient recognition sites. During the permeability studies, when compared with the non-imprinted membranes, the concentration of the ART in the receiving chamber was significantly higher than that of the ARE for both MMIM1 and MMIM0, demonstrating that the selective imprinted sites in the membrane facilitated the notably faster transport of ART compared to ARE. But, after 90 min, the value of ART/ARE toward MMIM0 decreased to 1.5, while the *β*_ART/ARE_ value was maintained at 5.0, which may be ascribed to the high-density effective recognition sites on the surface of MMIM1. It can be concluded that the selective sites of MIMI1 on the upper surface facilitated the transport of the ART. 

**Figure 4:**
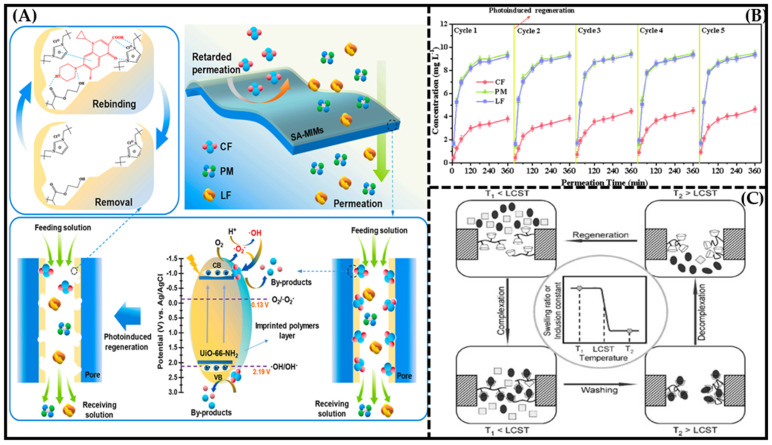
(**A**) Schematics of selective separation mechanism and photoinduced rapid regeneration process of SA−MIM. Reproduced with permission from [93]. Copyright 2022 Elesvier; (**B**) regeneration ability of SA−MIM tested by selective permeation experiment. Reproduced with permission from [93]. Copyright 2022 Elesvier; (**C**) schematic illustration of the proposed concept of a thermoresponsive membrane for chiral resolution and the membrane process. Reproduced with permission from [94]. Copyright 2008 Adv. Funct. Mater.

Kashani et al. [95] fabricated nanopore-molecularly-imprinted polymer membranes with average pore diameters of ca. 300–800 nm using the phase-inversion method for the selective separation of 2,4-dichlorophenoxyacetic acid, a toxic herbicide, from water. In their research, the authors compared the permselectivity of various imprinted membranes with the addition of different MIPs, as well as distinct pore sizes. For all the MIMs (MIM1, MIM2, and MIM3), the membrane permeability for 2,4-D was greater than that of POAc, according to the mechanism of facilitated permeation. However, among the fabricated MIMs, MIM-1, which had the smallest pore diameter (326 nm), had the highest separation factor. The differences in selective permeation performance between MIM1, MIM2, and MIM3 can be attributed to their surface characteristics. The MIM1 had a dense surface, which prevented the POAc molecules from passing through the membrane cavities due to the lack of complementarity between the imprinted cavities in the MIPs and the analog molecules. On the other hand, MIM2 and MIM3 had higher porosity and more cavities on their surfaces, allowing the POAc molecules to pass through the membrane, thereby reducing the selective permeation performances of these MIMs.

Subsequently, Meng et al. [76] prepared a ceramic alumina-imprinted membrane by introducing an ordered porous polymer network into the membrane’s interior with room-temperature ionic liquid (RTIL) as the pore template. Compared with the Al_2_O_3_ ceramic membrane, the imprinting polymer network filled all the pore channels of the Al_2_O_3_ ceramic membrane. In the permeation experiment, the results showed that CA–CIAM2 preferentially transported the SA molecules other than the template molecule (GA). This may be ascribed to the membrane structure of the CA–CIAM2 filling with a large number of hierarchical holes. Therefore, the template molecule (GA) can easily selectively bind to the functional groups in the recognition sites on the imprinted membrane. Meanwhile, due to the mismatch in size and shape between the SA molecules and the recognition cavity, the imprinted membrane exhibited limited recognition effects on the non-template molecules, and the SA molecules were effortlessly desorbed from the membrane. Consequently, there was a higher likelihood of the SA continuously transferring from one side of the composite membrane to the other. Thus, the imprinted membrane functioned as an adsorptive membrane. As a result, the transport mechanism for the permeation of the SA and GA towards the imprinted Al_2_O_3_ membrane agreed with the retarded permeation mechanism.

In a word, the membrane structure, pore-size distribution, and density of recognition sites in the final imprinted membrane collectively determine the mass-transfer mechanism of the membrane.

### 4.2. Regeneration Performance

As mentioned above, although it is of great importance to improve the permselectivity/flux of MIMs simultaneously, the drawback of the deterioration and ultimate loss of separation ability should not be overlooked. In the separation process, the specific binding sites in the MIM are often gradually occupied by print molecules as the operating time increases. Therefore, the regenerability of MIMs is an important indicator in practical applications. Currently, the most commonly regeneration method is to rinse the membrane with a specific solvent for a certain time. Table 4 summarizes the relevant regeneration performances of various MIMs. The table indicates that the acid-eluting agent is usually used to realize the adsorption/desorption cycles. All the MIMs reported in the table showed good regeneration performance.

Certainly, considering that the most common regeneration methods require the use of a large amount of organic solvent for long periods, some researchers explored some new approaches to membrane regeneration. For instance, Yang et al. [94] constructed a novel thermoresponsive membrane for chiral resolution based on molecular recognition of beta-cyclodextrin (β-CD) and thermosensitivity based on the phase transition of poly(N-isopropylacrylamide) (PNIPAM). Figure 4C illustrates the schematics of the thermoresponsive membrane for chiral resolution. In the membrane, β-CD molecules act as host molecules, and PNIPAM chains act as microenvironmental adjusters. When the membrane is operated at a temperature below the LCST, one of the two enantiomers is captured by β-CD molecules, while the other is permeated. When the complexation between β-CD and the captured molecules reaches equilibrium, a wash process is carried out to remove the uncaptured or free molecules. Next, the operation temperature is increased to above the LCST, the PNIPAM chains shrink, and the captured molecules on the β-CD decrease significantly; thus, the enantiomers are separated and the membrane is regenerated. This membrane-regeneration method is completely environmentally friendly and can be easily operated.

Another type [93] of membrane-regeneration method was reported by Xing et al., who fabricated a new molecularly imprinted membrane (SA-MIM) based on UiO-66-NH_2_ with photoinduced regeneration ability, which showed a long-lasting selective separation ability for ciprofloxacin (CF) using the photocatalytic activity of UiO-66-NH_2_ (Figure 4A). The process of photo-induced regeneration is extremely rapid, lasting about two hours, with an illumination time of 15 min. Figure 4B shows the regeneration ability of SA-MIM tested by a selective permeation experiment. The maximum permselectivity coefficients (β) of the SA-MIM after five cycles still reached 2.73 (β_CF/PM_) and 2.71 (β_CF/LF_), suggesting an excellent regenerative performance. Therefore, the photocatalytic properties of UiO-66-NH_2_ can make the SA-MIM have the ability of rapid regeneration.

In summary, we need to further explore greener, more efficient, and environmentally friendly regeneration methods for MIMs to enable their industrial-scale application as soon as possible.

### 4.3. Antifouling Performance

Fouling is the deposition of retained particles, colloids, macromolecules, salts, etc., at the membrane surface or inside the pore at the pore wall [101]. For almost all membrane-based liquid-separation processes, flux reduction due to fouling is the single most important problem affecting performance and economics [102]. The modification of the surface hydrophilicity of membranes can have a significant impact on their fouling-resistance properties. Increasing the hydrophilicity of a membrane’s surface can enhance its ability to resist fouling caused by proteins and other similar foulants.

Dong et al. [100] fabricated antifouling molecularly imprinted membranes by introducing PEI and DA for the selective separation and detection of lincomycin in milk pretreatment. In the experiment, the combined presence of PEI and DA had a synergistic impact on the enhancement of the hydrophilicity of the original PVDF membrane. This hydrophilic modification method can effectively improve the anti-pollution capacity of the LINMIM. In another study [103], one pot-economical approach was used to fabricate a MIM by using carbon-nanosphere sol as a coagulation bath during the phase-inversion process. A significant enhancement of the antifouling property was observed with CNS as the coagulation bath. In addition, different types of hydrophilic material have been introduced to membrane preparation to increase the hydrophilicity and roughness of surfaces, thus mitigating fouling, such as the use of Ag nanoparticles, GO, TiO_2_, and SiO_2_ enhance the anti-fouling performances of imprinted membranes [104,105].

Furthermore, researchers have also blended hydrophilic MIPs with polymer materials to achieve a higher-performance membranes. Yang et al. prepared polyethersulfone-ultrafiltration membranes modified with MAA-EGDMA-imprinted polymers for selective atrazine separation. In their research, a blend of imprinted polymers (MIP/NIP) was incorporated into a polyether–sulfone (PES)-doped solution to facilitate the separation of atrazine and mitigate the fouling on the PES filtration membrane. The addition of the MIP to the membrane proved its hydrophilicity and antifouling properties compared to the pristine PES membrane.

**Figure 5:**
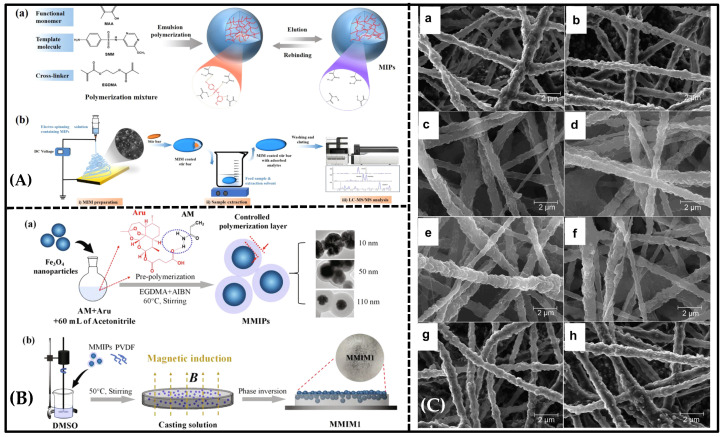
(**A**) (**a**) Preparation of MIPs by emulsion polymerization. (**b**) MIM-preparation--and-detection process. Reproduced with permission from [106]. Copyright 2021 Elsevier. (**B**) Schematic diagram of (**a**) the preparation process of magnetic molecularly imprinted polymer. (**b**) the preparation process of magnetically induced PVDF-based molecularly imprinted membrane. Reproduced with permission from [92]. Copyright 2020 Elsevier. (**C**) SEM images of nanofibrous membranes (**a**) F(mipB), (**b**) F(nipB), (**c**) F(mipT), (**d**) F(nipT), (**e**) F(mipBT3), (**f**) F(nipBT3), (**g**) F(mipB + mipT), (**h**) F(nipB + nipT). Reproduced with permission from [107]. Copyright 2014 Elsevier.

Based on the findings mentioned above and on previous studies, it can be reasonably inferred that the improved hydrophilicity of MIMs is the contributing factor to their superior antifouling performance.

As is known, beyond hydrophilicity, surface charge and roughness also play a crucial role in membrane fouling. The formation of a fouling-resistant coating on the membrane surface using blending and photochemical and chemical grafting is a highly efficient path to hampering the deposition of colloids. Membrane fouling is mitigated, but not totally eliminated [108]. Once fouling deposition has occurred, the efficacy of surface modification in preventing fouling diminishes. This suggests that there no membranes completely immune to fouling under any circumstances. It is essential to consider the integration of additional measures to prevent foulant deposition on membrane surfaces. This may involve incorporating other devices, such as specialized membrane-module designs or implementing effective membrane-cleaning techniques.

Therefore, the arduous task in our research is to design and prepare durable and anti-fouling molecularly imprinted membranes for practical applications.

## 5. Emerging Molecularly Imprinted Membranes in Separation

### 5.1. Molecularly Imprinted Nanofiber Membranes

Molecularly imprinted nanofiber membranes are expected to solve the trade-off relationship in membrane separation, since nanofiber membranes provide a greater surface area and higher porosity [81,109]. Electro-spun nanofiber membranes [110,111] exhibit notable attributes, such as their significant specific surface area, elevated porosity, and convenient modification capability. During the electrospinning process, the propulsion of nanofibers from a metal needle is achieved by harnessing the electrostatic forces arising from an externally applied high-voltage electrical field. These nanofibers are expelled through a spinneret filled with spinning fluid and subsequently deposited onto a collector surface [112]. This process allows the production of membranes with desirable properties. Chronakis initially described a straightforward approach to creating molecular-recognition sites within polymer nanofibers. A molecularly imprinted membrane via electrospinning was created by blending poly(ethylene terephthalate) (PET) and polyallylamine in a solution containing the template molecule, 2,4-dichlorophenoxyacetic acid (2,4-D) [81]. In this study, polyamine was utilized to incorporate functional groups that engage in interactions with the template molecule, while PET served as a supportive matrix. The involvement of dipole–dipole interactions between the π-electron systems of the benzene rings and the carbonyl groups in the PET backbone contributed to the preservation of recognition sites during the template-removal process. The research suggested that the molecular interaction between polyallylamine and the carboxyl group of the template molecule became dominant as the solvent completely evaporated at the final stage of the electrospinning process. The PET component contributed to additional interpolymer chain interactions, ensuring the fidelity of the binding sites and enhancing the strength of the fiber matrix (Figure 2C). In this study, suitable functional polymers were integrated with a supporting material through electrospinning to create template-specific binding sites. However, it is worth mentioning that the selection of functional polymers for imprinting in electrospinning is dependent on the template molecules, which imposes certain limitations on the development of electro-spun imprinted membranes.

Another method of MIM preparation involves the doping of a certain amount of MIP solution in the electrospinning solvent, ensuring that the MIP-based uniformly distributed nanofiber membrane can be obtained after the electro-spinning procedure. Cui et al. reported a morphology-controllable MIM, which they achieved by incorporating molecularly imprinted polymers (MIPs) into the electrospinning solution and utilizing the electrospinning technique (Figure 5A) [106]. In this research, MIPs were initially carried out using emulsion polymerization, with sulfamonomethoxine serving as the template molecule. The resulting MIM showed remarkable selectivity for sulfonamides, as evidenced by the selectivity factor β ranging from 2.3 to 2.7. Moreover, Wu et al. reported, for the first time, that MIMs containing different types of MIP-NPs can be used to realize the simultaneous extraction of both acidic and basic analytes with high selectivity (Figure 5C) [107]. The researchers developed a selective m-MISPE with multi-analyte selectivity and extracted trace BPA and TBZ from different vegetable and juice samples, which is a more effective and convenient method than the use of commercial C18/SCX sorbents. Electro-spun nano-MIMs with multi-analyte selectivity are expected to simplify analytical sample preparation, proving to be convenient tools for use in chemistry and biology laboratories.

**Figure 6:**
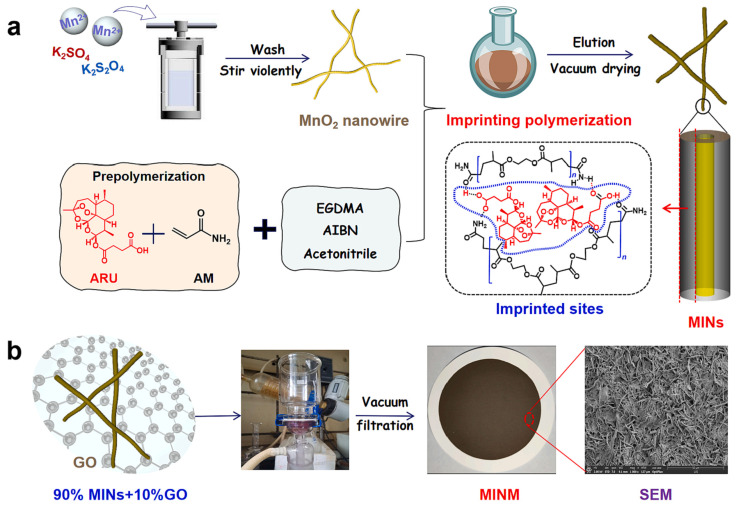
Schematic diagram of (**a**) the process of preparing molecularly imprinted polymer. (**b**) the process of preparing molecularly imprinted membrane Reproduced with permission from [36]. Copyright 2023 Elsevier.

Recently, some new molecularly imprinted nanofiber membranes were developed for higher separation ability. By vacuum-filtering imprinted manganese dioxide (MnO_2_) nanowires and graphene-oxide nanosheets onto the surface of a polyvinylidene fluoride (PVDF) membrane, Meng et al. first fabricated a hydrophilic artemisinin (ART)-imprinted MnO_2_ nanowire “coating” membrane (MINM) for selective ART separation [76]. The MINM was fabricated to break the bottleneck of the traditional MIM and had ultrahigh selectivity and adsorption capacity. The mechanism through which the ART is adsorbed by the MINM was investigated using the ATR FT-IR dynamic spectrum. The study revealed the in situ formation of hydrogen bonds between the ART and the MINM, providing insights into the adsorption process (Figure 6) [36].

### 5.2. New Phase-Inversion Molecularly Imprinted Membrane

Among numerous approaches, the phase-inversion technique is an alternative approach to the preparation of MIMs [113]. The presence of template molecules during the membrane-forming step encourages the formation of specific recognition sites in the membrane matrix. However, traditional MIMs exhibit poor selectivity due to the swelling of the membrane in the presence of certain solvents, which weakens the affinity of the recognition sites towards template molecules [82]. To overcome this limitation, the phase-inversion method was developed, in which pre-synthesized MIPs particles are embedded into the membrane matrix. However, this method has its own drawbacks, as it limits the number of available recognition sites and affects the separation efficiency of MIMs to some extent. Recently, Bai et al. [92] reported a new magnetic molecularly imprinted membrane (MMIM) with imprinted sites located and dispersed on the membrane’s upper surface via the phase-inversion method, using the magnetic field’s force for the selective separation of artemisinin (ART) and artemether (ARE) (Figure 5B). Before the phase inversion, the pre-synthesized magnetic MIPs in the polymer-casting solution migrated to the surface of the solution in the magnetic field, and then the casting solution was frozen to form an ordered macroporous lattice structure inside the membrane, and the membrane flux increased. This MIM can offer several advantages that help to overcome the drawbacks of MIMs produced through conventional phase inversion (1) The pre-synthesis of rigid MIPs effectively mitigates the swelling of polymeric materials during the phase-inversion-synthesis process. (2) The accumulation and dispersion of the majority of the MIPs on the upper surface of the membrane significantly increase the number of recognition sites, preventing the undesired embedding of polymers in the imprinted sites. In comparison to the control membrane (MMIM0) without magnetic guidance, the magnetic molecularly imprinted membrane (MMIM1) demonstrated remarkable improvement in recognized adsorption and achieved the highly efficient separation of ART and ARE.

**Figure 7:**
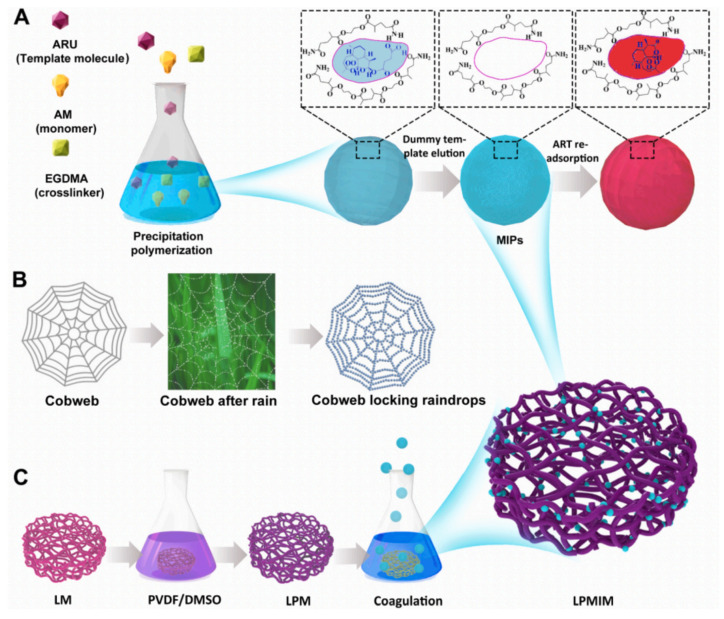
The schematic diagram (**A**) illustrates the synthesis, elution, and re-adsorption process of MIPs; The sketch map (**B**) depicts the concept of cobweb-locking raindrops; The process diagram (**C**) illustrates the preparation of the LPMIM. Reproduced with permission from [114]. Copyright 2022 Elsevier.

Furthermore, Bai et al. introduced a novel “delayed phase inversion” strategy for the spontaneous anchoring of ART-based MIPs onto the surfaces of a PVDF-modified loofah framework (LP), enabling the construction of a 3D porous LP-based molecularly imprinted membrane (LPMIM) for the highly efficient and selective separation of ART, as shown in Figure 7 [114]. The PVDF-modified loofah (LP) membrane was initially prepared using a lucerne fiber backbone immersed in a polyvinylidene fluoride (PVDF) casting solution, serving as the hydrophobic fibrous skeleton, or “cobweb.” Subsequently, MIPs, symbolizing “raindrops,” were dispersed in a water/ethanol solution. Upon the immersion of the LP membrane in the MIP/water/ethanol mixture, the MIPs spontaneously migrated to the fiber surfaces of the LP membrane, facilitated by the hydrophobic–hydrophobic interaction between the MIPs and the PVDF on the LP surfaces. This process resulted in the formation of the LP-supporting MIP membrane (LPMIM), in which numerous MIPs were clearly locked and dispersed on the LP surfaces, leading to high adsorption capacity. The delayed phase-inversion process facilitated the spontaneous anchoring and dispersion of the MIPs on the internal surfaces of the PVDF-modified loofah matrix, enabling the formation of the MIM. 

These studies have significantly driven the new direction of the development of the phase-inversion method for preparing imprinted membranes.

### 5.3. Metal–Organic-Framework-Material-Based Molecularly Imprinted Membrane

**Figure 8:**
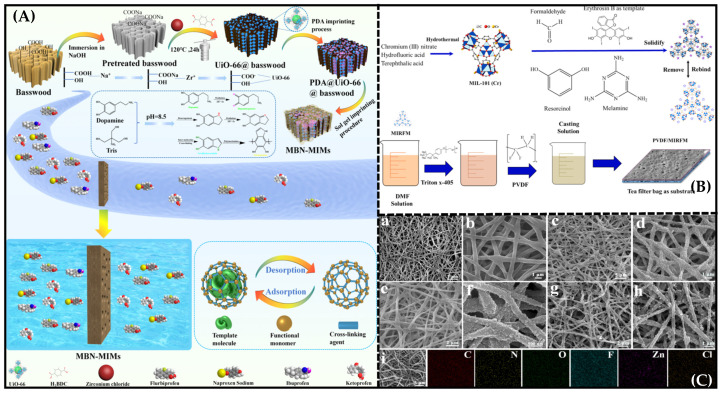
(**A**) Steps in the synthesis of BD-MOF/BMs. Reproduced with permission from [118]. Copyright 2023 American Chemical Society; (**B**) Schematic description of the formation and mechanism of the composite membrane. Reproduced with permission from [117]. Copyright 2022 Elesvier; (**C**) SEM images of the fabricated nanofibrous membrane at each stage: (a,b) PVDF/PVA, (c,d) ZIF-8@PVDF/PVA, (e,f) ZIF-V@PVDF/PVA, (g,h) A-MNMs; (i) EDX elemental mapping images of A-MNMs. Reproduced with permission from [119]. Copyright 2021 American Chemical Society.

Metal–organic frameworks (MOF) are porous crystalline materials with extended structures formed by the self-assembly of metal ions with organic ligands [115,116]. These coordinated polymers have the following characteristics =: (1) relatively large specific surface areas; (2) high thermal stability; (3) porous, adjustable apertures; (4) structural and functional diversity; (5) wide use in various fields. Bakhshizadeh et al. [117] reported a new imprinted membrane based on PVDF blending with hydrophilic molecularly imprinted MIL-101 (Cr) for the efficient selective removal of dye, as shown in Figure 8B. The contact-angle test proved that MIL-101 (Cr) can improve the surface hydrophilicity of the composite membrane and the removal selectivity of erythrosine, indicating that these MIMs are promising adsorption membranes for wastewater treatment with high selectivity, renewability and suitability for large-scale production.

Furthermore, Ma et al. reported biomimetic dual-layer imprinted UiO-66-based basswood membranes for ibuprofen recognition and separation, as depicted in Figure 8A [118]. Abundant ibuprofen-imprinted sites were obtained in the MIM based on the in situ growth and dual-imprinted processes of UiO-66, the uniform distribution of the UiO-66 in in the Basswood channel, increases in the specific surface area of the MIM, which resulted in the acquisition of abundant ibuprofen-imprinting sites, and a large and increasing number of imprinting sites, which enabled the prepared MIM to exhibit particularly high recombination capacity (120.6 mg g^−1^) and fast adsorption kinetics.

Xing et al. [119] successfully prepared novel atrazine (ATZ)-based molecularly imprinted nanofiber membranes and a molecular organic framework (MOF)-based sticky bead structure for the selective separation of ATZ. The most critical feature of the design was that MOF nanocrystals were uniformly assembled on the PVDF/PVA blended nanofibrous membrane’s surface through a solution-contra-diffusion method, and the specific recognition sites were efficiently constructed on the surfaces and pores of the MOF by using a surface imprinting strategy (Figure 8C). Compared with other MIMs, the molecularly imprinted nanofibrous membranes, characterized by a spider-web-like structure, exhibited improved rebinding capacity (37.62 mg g^−1^) and high permselectivity (the permselectivity factors *β* were 4.21 and 4.31) towards ATZ.

The MOF material introduced in the preparation of the MIM proved efficient for selective separation with higher separation capacity and better selectivity. The MOF-based MIM thus showed tremendous potential for large-scale selective separation industrial applications.

## 6. Conclusions and Perspectives

Although MIMs have been used in many fields, these MIMs represent only a small fraction of the vast array of MIMs with selective separation capabilities that have been reported. In particular, the permselectivity and the flux often have a trade-off relationship in membrane separation. However, neither the flux nor the permselectivity of these membranes are sufficient for industrial applications. To achieve higher flux and permselectivity, there is a need for MIMs possessing higher numbers of effective recognition sites and greater porosity. Moreover, to evaluate the feasibility of the industrial use of these membranes, a broader consideration of anti-fouling and regeneration performance is crucial.

It was found that, in addition to constructing the effective high-density recognition sites in the MIM, the strong matching of imprinted cavities is of paramount importance in solving the permselectivity/flux trade-off relationship. Similarly, the membrane structure, pore-size distribution, and recognition sites in the final imprinted membrane collectively determine the selective transport mechanism of the MIM. Recently, emerging forms of MIM, including molecularly imprinted nanofiber membranes, new phase-inversion MIMs, and metal–organic-framework-material-based MIMs have brought new prospects for the selective separation application of MIMs. This is especially true for MOF-based imprinted membranes, which can provide a large surface area and high porosity, as well as regular pore sizes. The former contributes to the enhancement of selectivity through the numerous imprinted sites uniformly fixed on the surface of the MOF surface, and the latter contributes to an increase in the diffusivity of the permeant. However, a possible breakthrough in solving the flux/permselectivity trade-off in MOF-based MIM separation may be provided by free-standing nanofibrous imprinted membranes fabricated by a more efficient method than electrospray deposition. Currently, the preparation of MOF-coated electro-spun MIMs always involves two approaches: blending electro-spinning and surface coating. The advantage of the blending of MOF-based MIPs with electro-spun nanofibers is that MOF-based MIPs can be loaded in the fibers steadily. However, they face the same challenge as composite beads, in that most of the MOF-based MIPs are inside the naonofibers, which impedes the contact between the MOF-based MIPs and the target molecules. The advantage of MOF-coated-MIP-electro-spun MIMs is that the nanofiber-imprinted membranes have more accessible adsorption sites for the target molecules. However, the imprinting process is uncontrollable, which causes the uneven distribution of imprinted sites, and different pore sizes decrease the separation effect.

Our suggested methods for further enhancing the membrane performances of MOF-based MIMs are as follows: (1) the localization of the molecular-recognition sites on the surfaces of MOF-coated nanofibers, which can be achieved through controlled polymerization reactions; (2) the construction of free-standing MIMs by connecting a monodisperse imprinted nanofiber through chemical bonds, such as click-on chemistry, which can better control the flux of the membrane.

## Figures and Tables

**Table 1:** The selection coefficient (THO / CAF) of Tho printing-and-dyeing film obtained at different coagulation temperatures. Reproduced with permission from [35]. Copyright 1997 Langmuir.

Coagulation Temp (°C)	[Sb]_T_ (µmol/g of Membrane)	[Sb]_C_ (µmol/g of Membrane)	α (THO/CAF)
10	1.25	0.024	52
15	0.85	0.030	28
30	0.48	0.026	18
40	0.26	0.023	11

**Table 3:** Comparison of previous studies on enantioselective membranes.

Number	Membrane	Concentration (mmol/L)	Flux (mg/cm^2^ × min)	α	Ref.
1	Supported liquid membrane	17.9	2.76 × 10^−5^	2	[85]
2	Supported liquid membrane	29.7	8.76 × 10^−4^	2.3	[86]
3	Supported liquid membrane	40	1.01 × 10^−5^	1.2	[87]
4	Porous ceramic disc and hollow-fiber organic membrane	44.74	6 × 10^−5^	15	[87]
5	Supported liquid membrane	44.74	6 × 10^−7^	2	[88]
6	Molecularly imprinted cellulose membrane	10	0.0198	7.3	[89]
7	Molecularly imprinted nano-channel membrane	10	0.070	8.7	[89]

**Table 4:** Comparison of regeneration performances of several MIMs.

Membrane	Split Object	Elution Solvent	Regeneration Times	Ref.
Antibacterial, high-flux, and 3D porous molecularly imprinted nanocomposite-sponge membranes	Emodining from analogues	A mixture of methanol and acetic acid (95:5, *v*/*v*)	10	[96]
Highly selective cellulose acetate (CA) blend imprinted membranes for salicylic acid (SA)	Template SA	A methanol/acetic acid (9:1, *v*/*v*) mixed solvent	5	[97]
Irregular-dot -array nanocomposite bisphenol A (BPA)-molecularly-imprinted membranes	Bisphenol A	A mixture of methanol and HAc (95:5, *v*/*v*)	10	[98]
Molecularly imprinted polymer (MIP) photonic film	Testosterone	A methanol/acetic acid (9:1, *v*/*v*) mixed solvent	6	[99]
Solvent-driven controllable molecularly imprinted membranes	Bisphenol A	100% MeOH	8	[93]
Lincomycin molecularly imprinted membrane	Lincomycin	A mixture ofmethanol and acetic acid (95:5, *v*/*v*)	10	[100]

## Data Availability

Data is contained within the article.
